# Supplementary material for: Consequences of heterogeneity in aging: parental age at death predicts midlife all-cause mortality and hospitalization in a Swedish national birth cohort
Source: BMC Geriatr. 2024 Feb 29;24:207. doi: 10.1186/s12877-024-04786-9 (PMC10903026; doi:10.1186/s12877-024-04786-9)
Supplement: Supplementary file 1 — Additional file 1: Figure S1. Description of the study sample selection. Figure S2. Cumulative hazards of index persons’ all-cause mortality, stratified by parental attained age based on chronological age (n= 89 688). Figure S3. Cumulative hazards of index persons’ hospitalizations, stratified by parental attained age based on chronological age (n= 89 688). Figure S4. Cumulative hazards of index persons’ all-cause mortality, stratified by quartiles of average parental attained age where the lower threshold for parental age at death was set to 65. (N= 75274). Figure S5. Cumulative hazards of index persons’ hospitalizations, stratified by quartiles of average parental attained age where the lower threshold for parental age at death was set to 65. (N= 75274). Figure S6. Cumulative hazards of index persons’ all-cause mortality, stratified by quartiles of maternal and paternal attained age on the total population (n= 89 688). Figure S7. Cumulative hazards of index persons’ hospitalizations, stratified by quartiles of paternal attained age on the total population (n= 89688). Figure S8. Cumulative hazards of index persons’ hospitalizations, stratified by quartiles of maternal attained age on the total population (n= 89688). Figure S9. Hazard rate ratios for stratified regression models using paternal and maternal attained age as exposure variable. Figure S10. Cumulative hazards of index persons’ hospitalizations, stratified by quartiles of parents attained age, men (n= 46048). Figure S11. Cumulative hazards of index persons’ hospitalizations, stratified by quartiles of parents attained age, women (n= 43640). [file 12877_2024_4786_MOESM1_ESM.pdf]

Supplemental material for: Consequences of Heterogeneity in Aging: Parental Age at Death Predicts Midlife All-Cause Mortality and Hospitalization in a Swedish National Birth Cohort

Anna Thalén and Anders Ledberg

Department of Public Health Sciences, Stockholm University
SE-106 91 Stockholm, Sweden
anders.ledberg@su.se or anders.ledberg@gmail.com

January 26, 2024

Contents

List of Figures

S1 Description of the study sample selection. 3
S2 Cumulative hazards of index persons' all-cause mortality, stratified by parental attained age based on chronological age (n= 89 688). 4
S3 Cumulative hazards of index persons' hospitalizations, stratified by parental attained age based on chronological age (n= 89 688). 5
S4 Cumulative hazards of index persons' all-cause mortality, stratified by quartiles of average parental attained age where the lower threshold for parental age at death was set to 65. (N= 75274) . . 6
S5 Cumulative hazards of index persons' hospitalizations, stratified by quartiles of average parental attained age where the lower threshold for parental age at death was set to 65. (N= 75274) . . 7
S6 Cumulative hazards of index persons' all-cause mortality, stratified by quartiles of maternal and paternal attained age on the total population (n= 89 688). 8
S7 Cumulative hazards of index persons' hospitalizations, stratified by quartiles of paternal attained age on the total population (n= 89688). 9
S8 Cumulative hazards of index persons' hospitalizations, stratified by quartiles of maternal attained age on the total population (n= 89688). 10

S9 Hazard rate ratios for stratified regression models using paternal and maternal attained age as exposure variable. 11
S10 Cumulative hazards of index persons' hospitalizations, stratified by quartiles of parents attained age, men (n= 46048). 12
S11 Cumulative hazards of index persons' hospitalizations, stratified by quartiles of parents attained age, women (n= 43640). 13

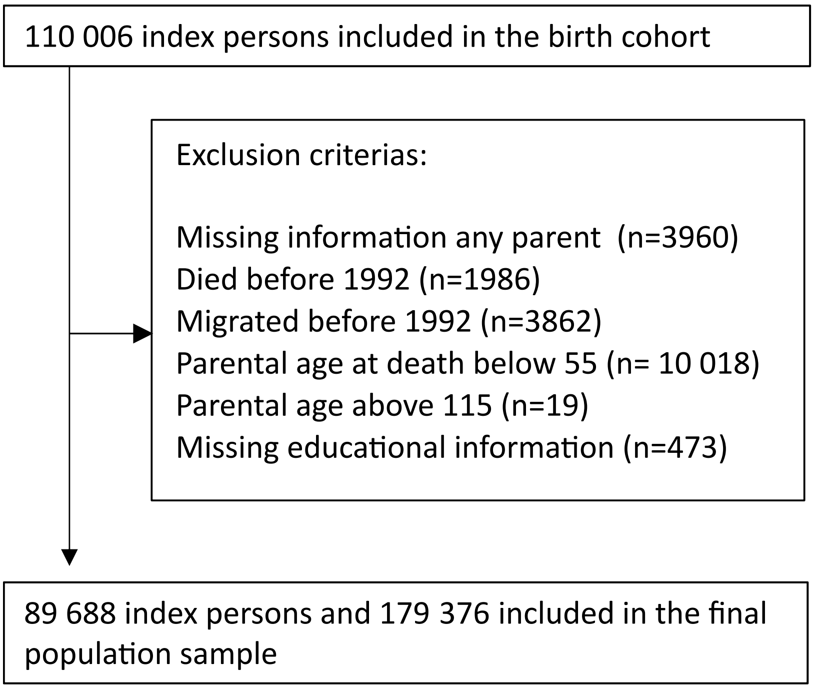

Figure S1: Description of the study sample selection.

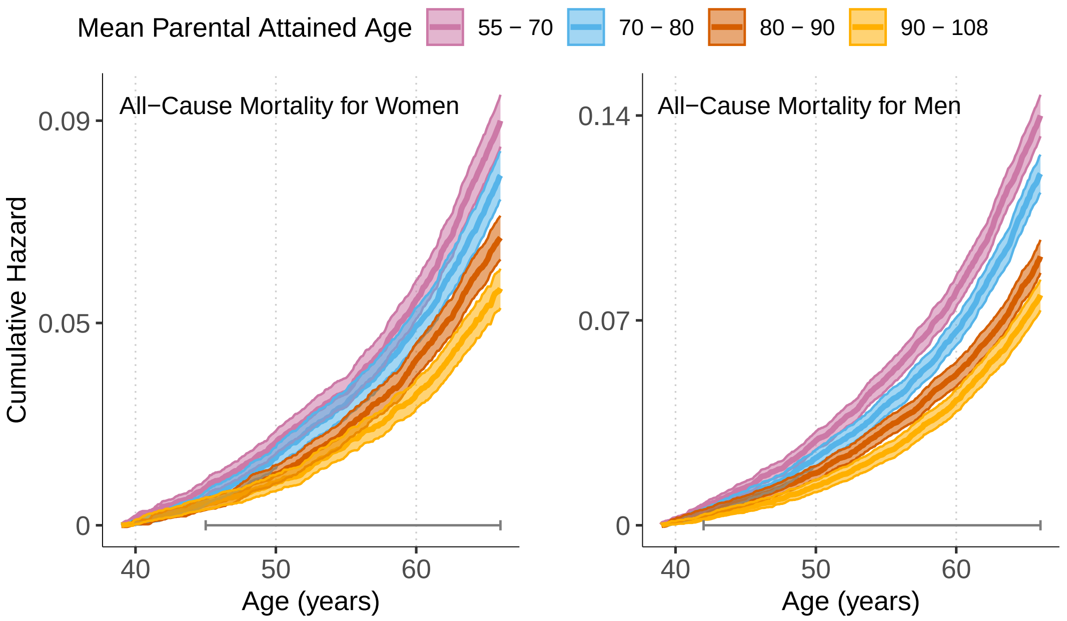

Figure S2: Cumulative hazards of index persons' all-cause mortality, stratified by parental attained age based on chronological age (n= 89 688).

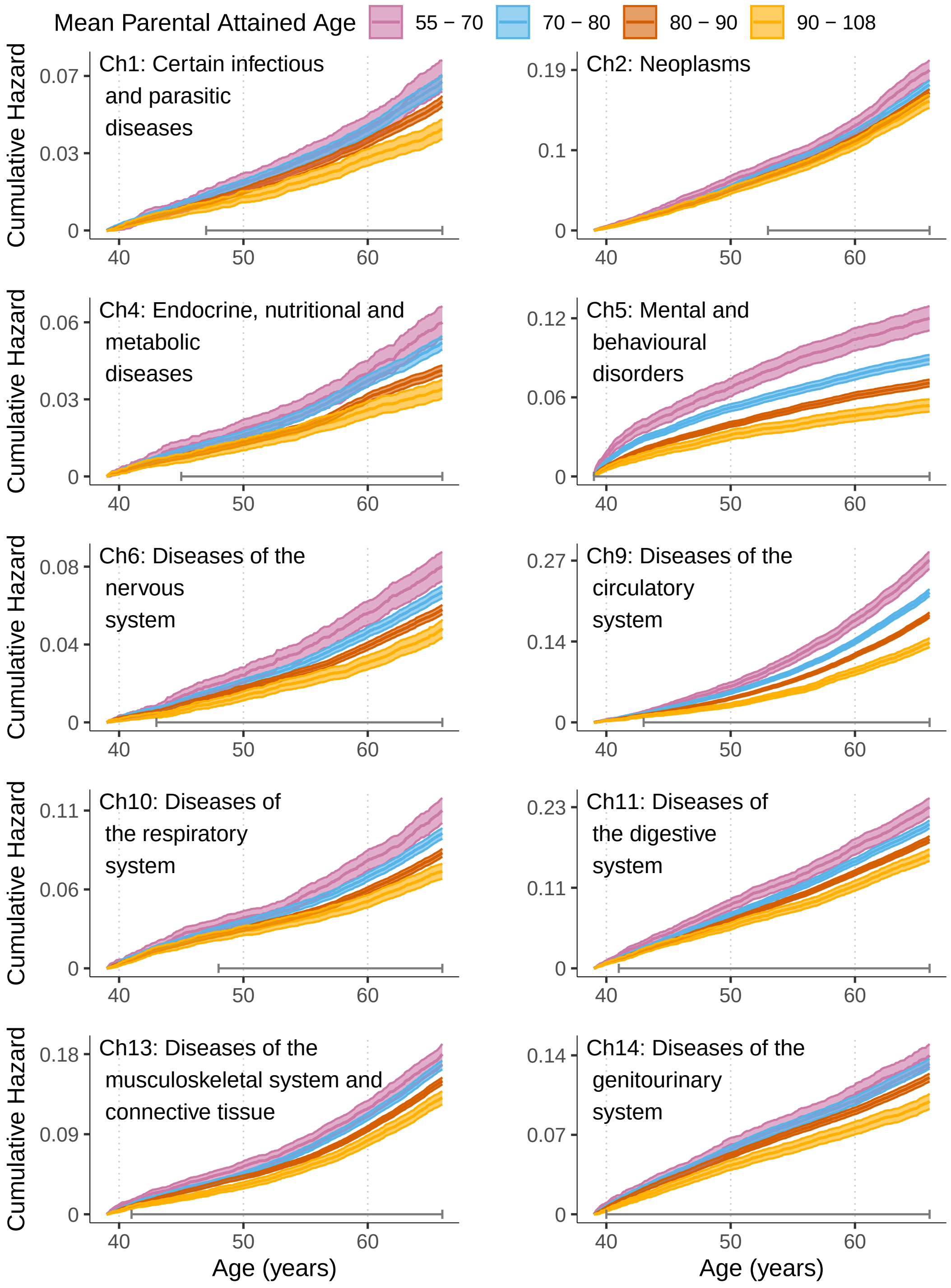

Figure S3: Cumulative hazards of index persons' hospitalizations, stratified by parental attained age based on chronological age (n= 89 688).

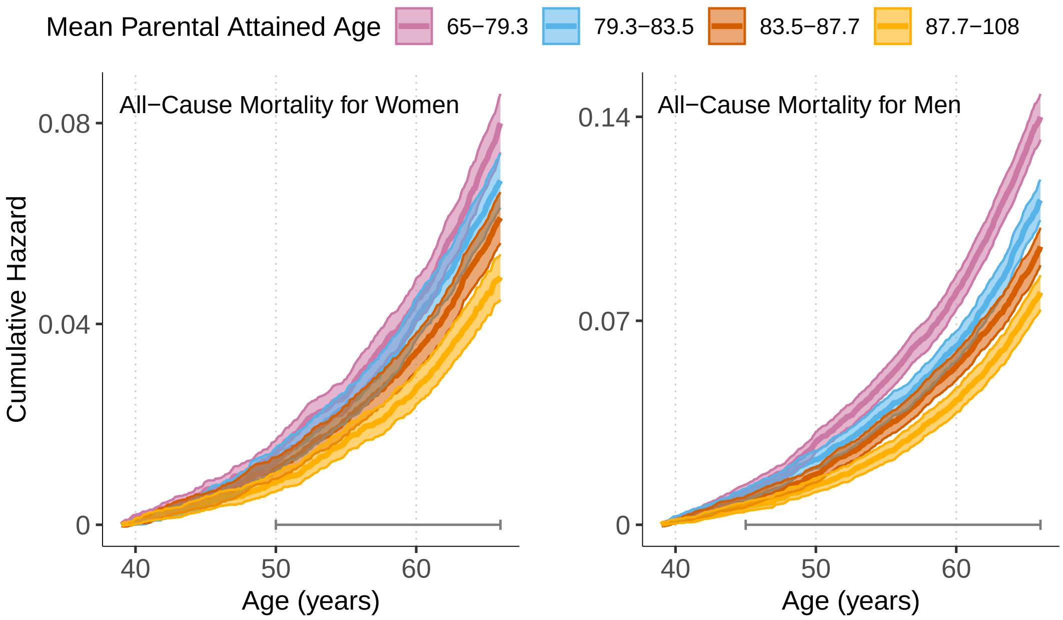

Figure S4: Cumulative hazards of index persons' all-cause mortality, stratified by quartiles of average parental attained age where the lower threshold for parental age at death was set to 65. (N= 75274)

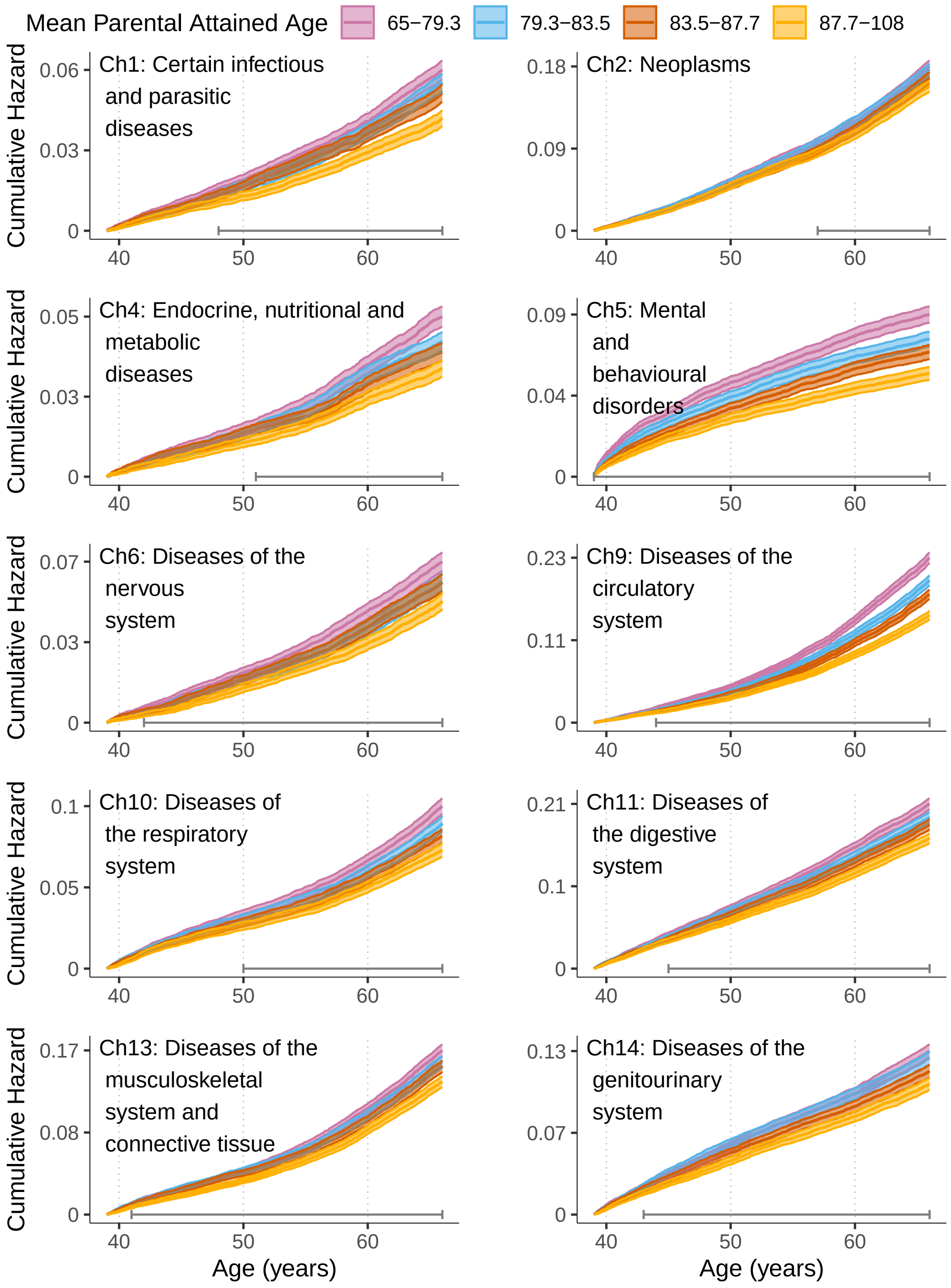

Figure S5: Cumulative hazards of index persons' hospitalizations, stratified by quartiles of average parental attained age where the lower threshold for parental age at death was set to 65. (N= 75274)

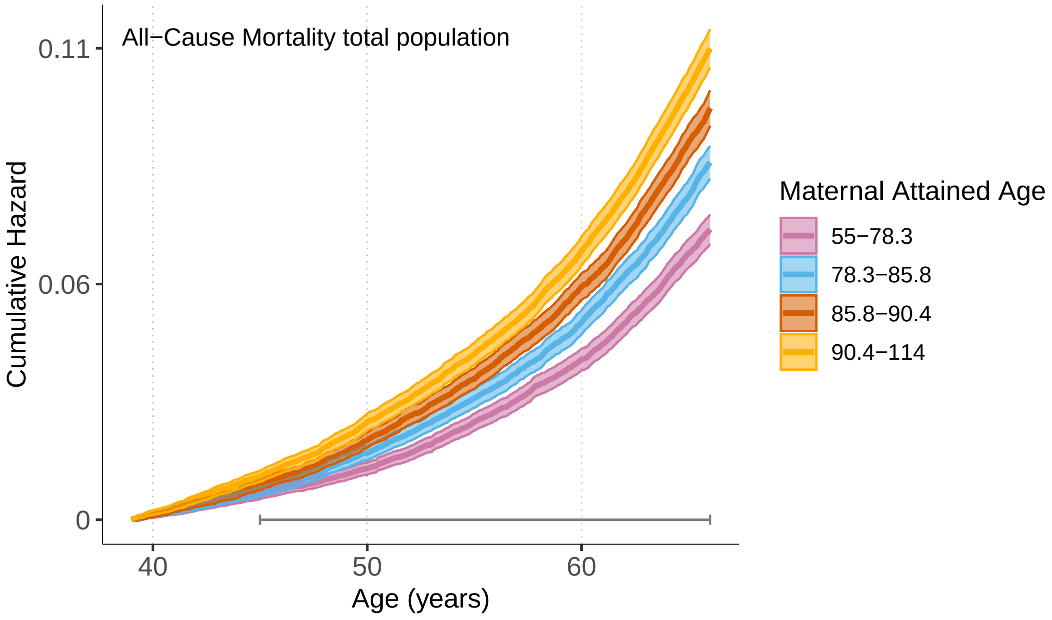

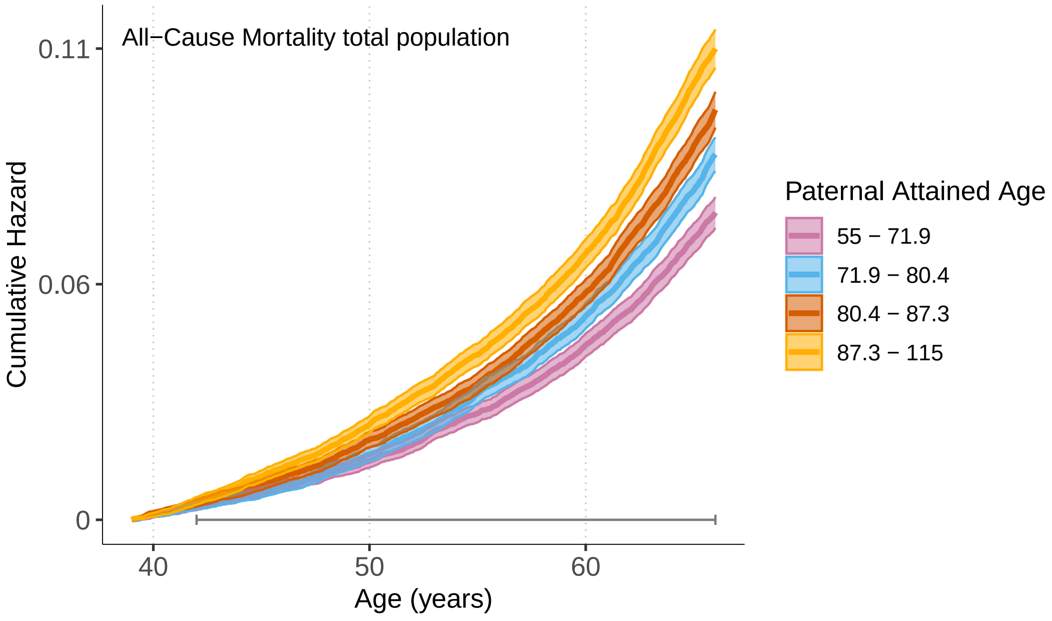

Figure S6: Cumulative hazards of index persons' all-cause mortality, stratified by quartiles of maternal and paternal attained age on the total population (n= 89 688).

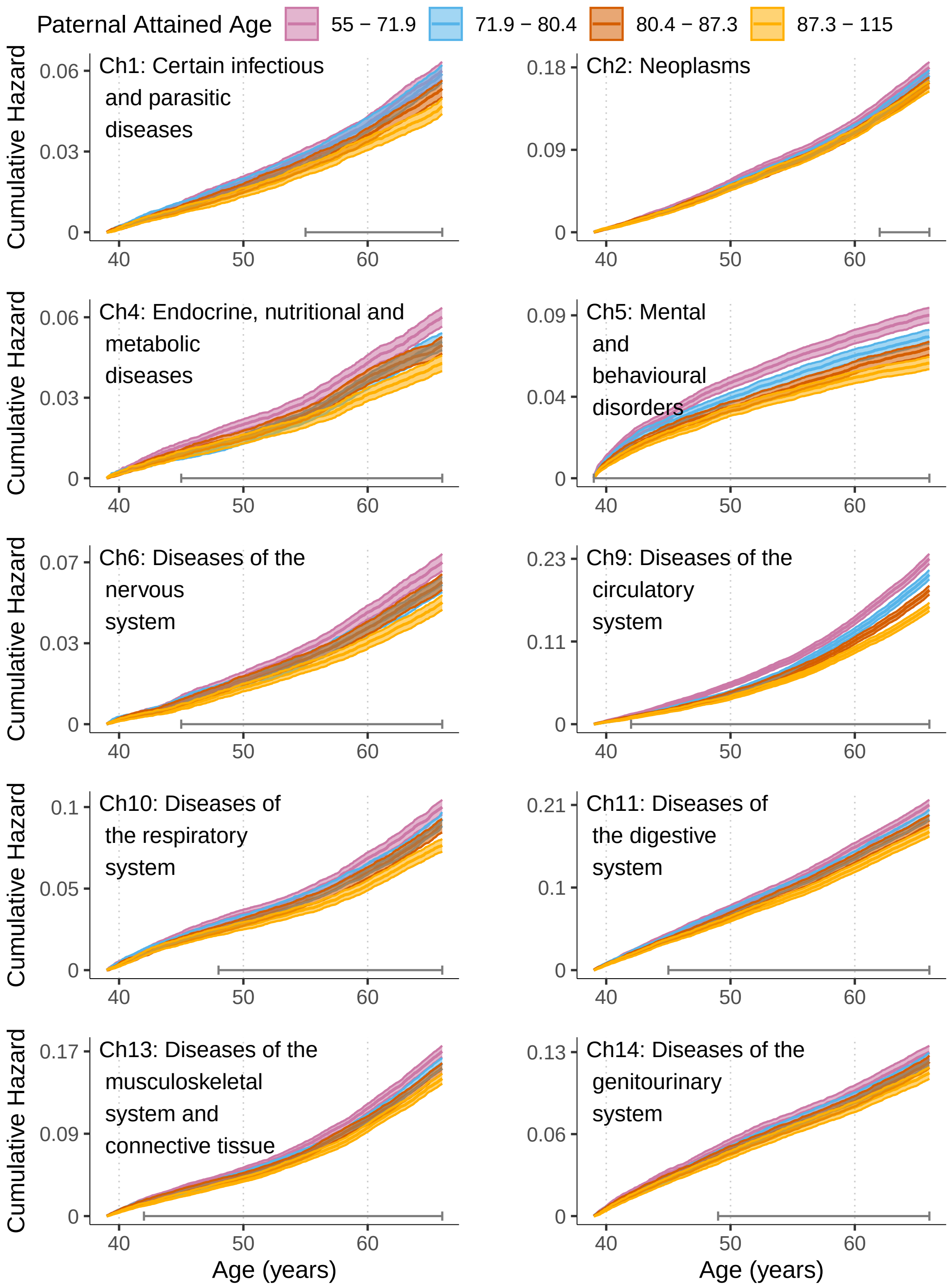

Figure S7: Cumulative hazards of index persons' hospitalizations, stratified by quartiles of paternal attained age on the total population (n= 89688).

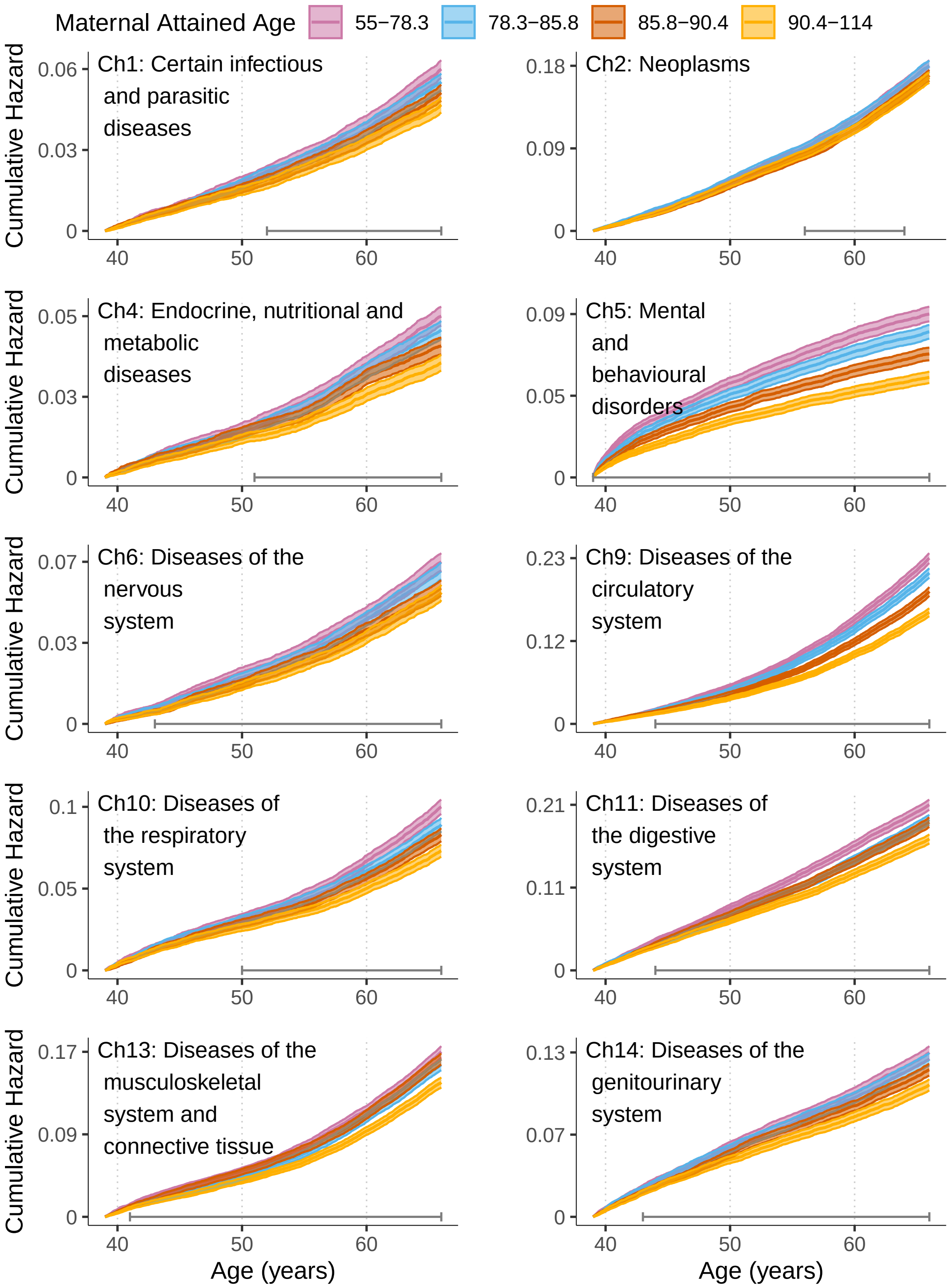

Figure S8: Cumulative hazards of index persons' hospitalizations, stratified by quartiles of maternal attained age on the total population (n= 89688).

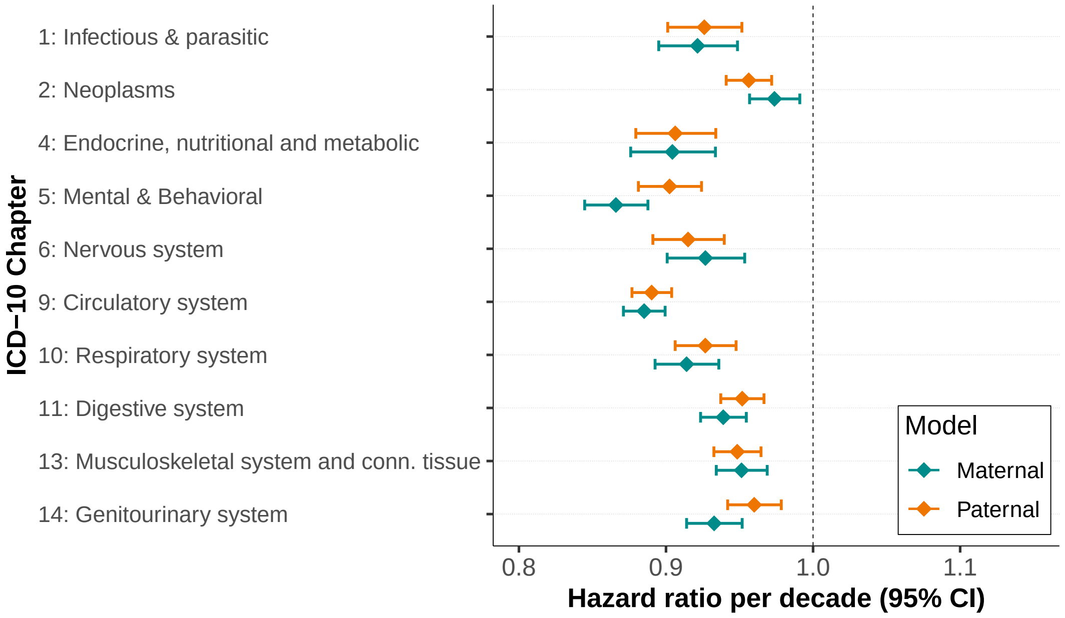

Figure S9: Hazard rate ratios for stratified regression models using paternal and maternal attained age as exposure variable.

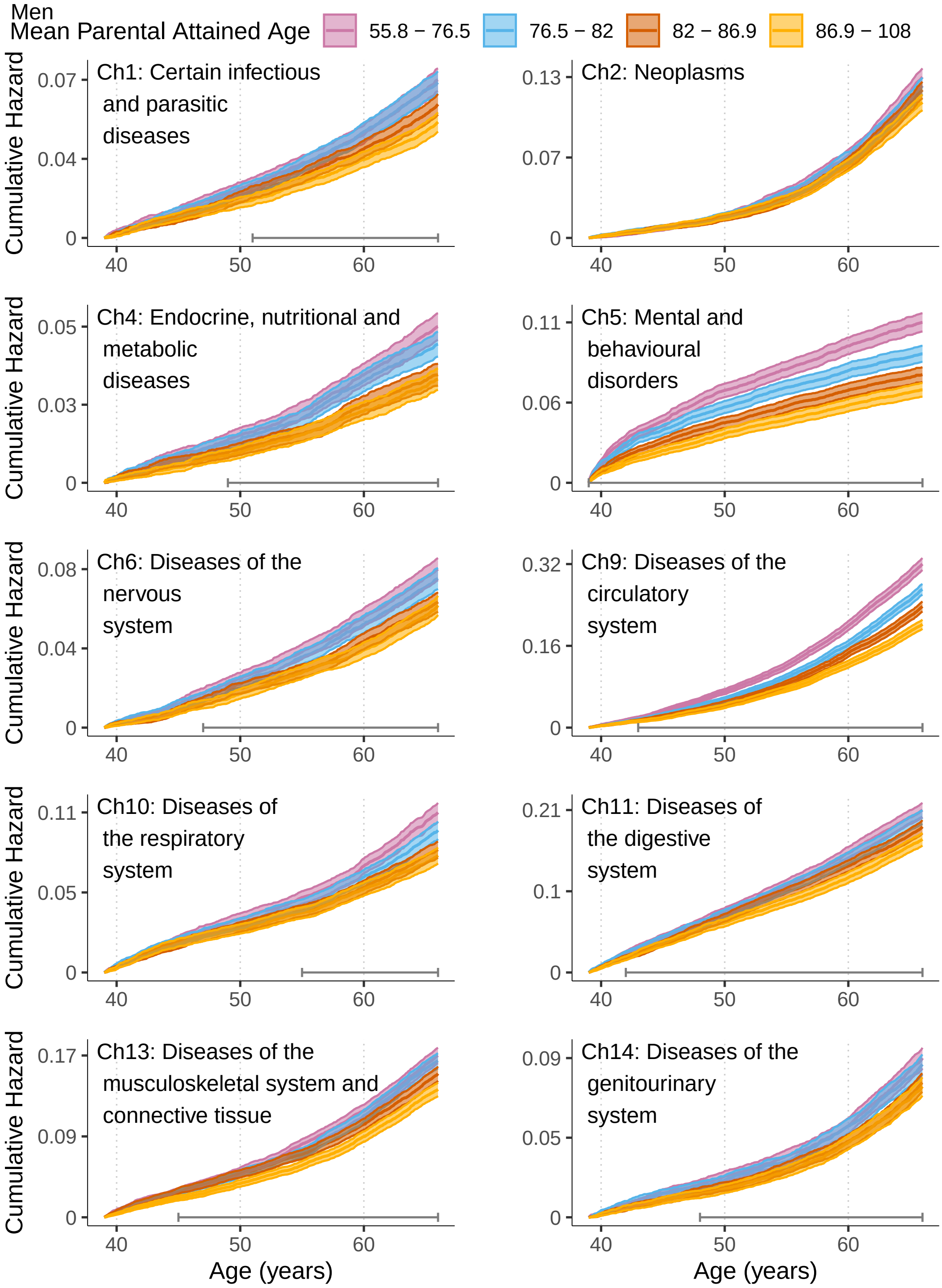

Figure S10: Cumulative hazards of index persons' hospitalizations, stratified by quartiles of parents attained age, men (n= 46048).

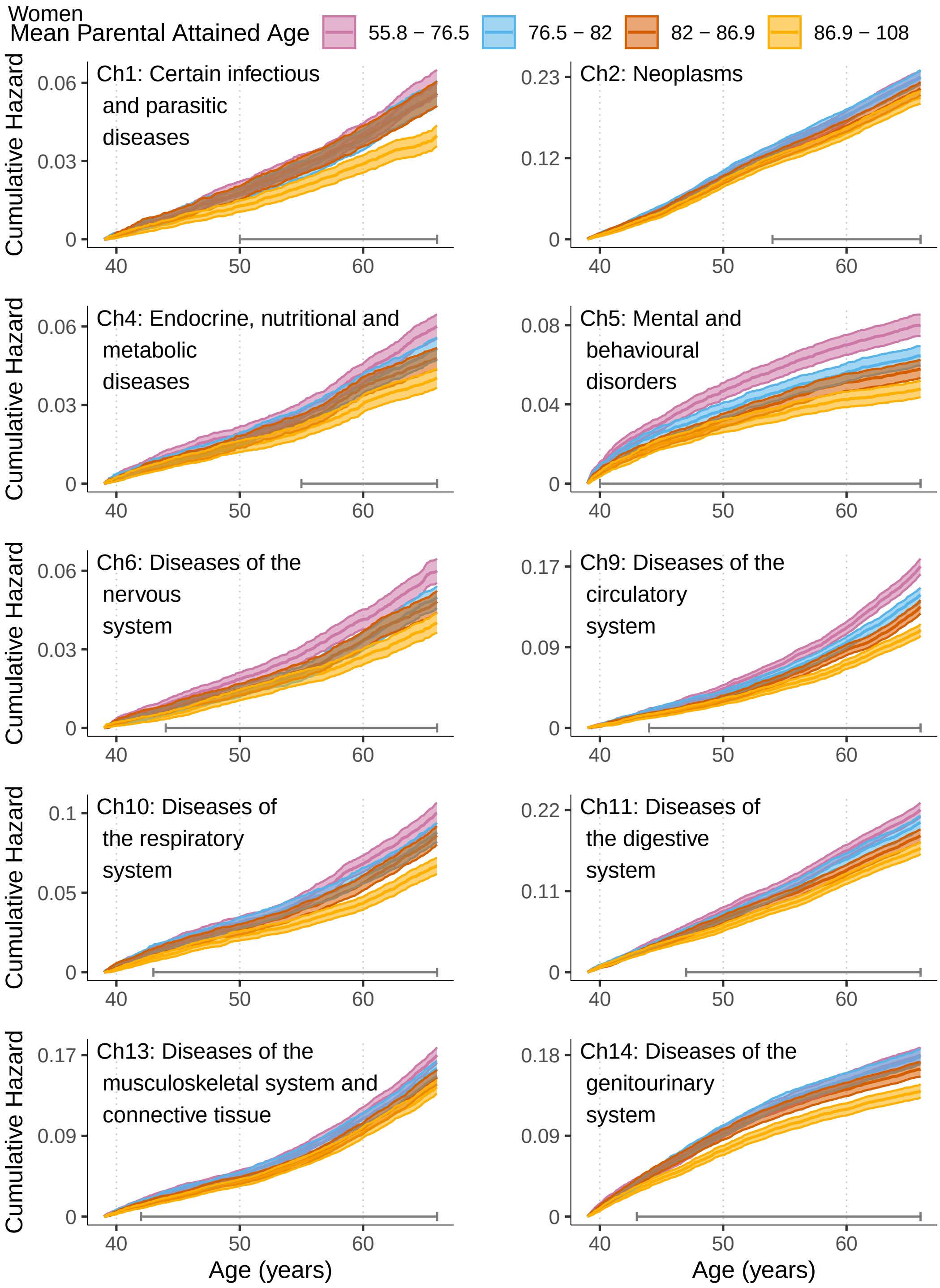

Figure S11: Cumulative hazards of index persons' hospitalizations, stratified by quartiles of parents attained age, women (n= 43640).
